# Metallic-mean quasicrystals as aperiodic approximants of periodic crystals

**DOI:** 10.1038/s41467-019-12147-z

**Published:** 2019-09-17

**Authors:** Joichiro Nakakura, Primož Ziherl, Junichi Matsuzawa, Tomonari Dotera

**Affiliations:** 1 Department of Physics, Kindai University, 3-4-1 Kowakae, Higashi-Osaka, 577-8502 Japan; 2 Faculty of Mathematics and Physics, University of Ljubljana, Jadranska 19, SI-1000 Ljubljana, Slovenia; 3 Jožef Stefan Institute, Jamova 39, SI-1000 Ljubljana, Slovenia; 4 Department of Mathematics, Nara Women’s University, Kitauoya-Nishimachi, Nara 630-8506 Japan

**Keywords:** Theory and computation, Structure of solids and liquids

## Abstract

Ever since the discovery of quasicrystals, periodic approximants of these aperiodic structures constitute a very useful experimental and theoretical device. Characterized by packing motifs typical for quasicrystals arranged in large unit cells, these approximants bridge the gap between periodic and aperiodic positional order. Here we propose a class of sequences of 2-D quasicrystals that consist of increasingly larger periodic domains and are marked by an ever more pronounced periodicity, thereby representing aperiodic approximants of a periodic crystal. Consisting of small and large triangles and rectangles, these tilings are based on the metallic means of multiples of 3, have a 6-fold rotational symmetry, and can be viewed as a projection of a non-cubic 4-D superspace lattice. Together with the non-metallic-mean three-tile hexagonal tilings, they provide a comprehensive theoretical framework for the complex structures seen, e.g., in some binary nanoparticles, oxide films, and intermetallic alloys.

## Abstract (web summary)

Quasicrystals differ from traditional incommensurate structures because they have non-crystallographic rotational symmetries. Here the authors introduce a scheme to produce metallic-mean quasicrystals in two dimensions with 6-fold rotational symmetry that can be seen as approximant to periodic tilings.

## Introduction

The once-traditional understanding of positional order in condensed matter, which postulates translational periodicity, permits only 2-, 3-, 4-, and 6-fold rotational symmetry. In 1984, this view was challenged by the observation of an icosahedral symmetry in an Al-Mn alloy^1^ immediately backed up theoretically by the now well-accepted existence of quasiperiodic positional order, which are essential for a sharp diffraction pattern, in aperiodic quasicrystals (QCs), such as the Penrose tiling^2^. In turn, the discovery of QCs offered a new perspective on complex crystalline lattices with large unit cells such as the Frank-Kasper phases^3^ (and tetrahedrally close-packed structures^4^ in general) and periodic crystals of large near-icosahedral clusters such as the Mackay icosahedra^5^, some of which were found to bear considerable similarity with QCs and are thus referred to as QC approximants^6,7^.

Approximants are among the cornerstones of QC science^8,9^. In experiments, they are typically seen at a somewhat different temperature, pressure, composition, etc. than QCs^10–15^ and they signal the proximity of QCs^16–19^, whereas theoretical studies of approximants allow one to explore structures that approach QCs in terms of both local arrangement of particles and material properties by using the mathematical apparatus developed for periodic crystals^20–22^. Approximants show that the simple, small-unit-cell periodic crystals and QCs are connected by a spectrum of structures that interpolate between the two extremes. This unified view of periodic crystals and QCs is further supported by the higher-dimensional analysis where both are seen as projections of a hyperdimensional lattice onto the physical space with rational and irrational tangents^8,9^.

**Fig. 1:**
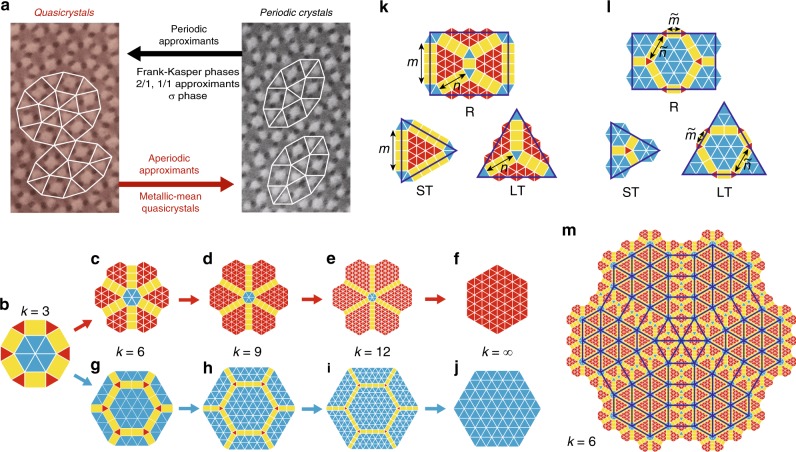
Metallic-mean aperiodic approximants. **a** Schematic showing the role of aperiodic approximants as a link between QCs and periodic crystals illustrated by bright-field transmission electron micrographs of dodecagonal QCs and (3^2^.4.3.4) Archimedean tiling observed in star terpolymer-homopolymer blend used in ref. ^17^; the latter consists exclusively of the rugby-ball local motifs (outline) whereas the former features various local motifs including the dodecagonal wheel. **b** First-generation bronze-mean *k* = 3 (*n* = 1, *m* = 1) tiling. **c**–**f** First-generation type IA tilings with *k* = 6 (*n* = 3, *m* = 4), *k* = 9 (*n* = 5, *m* = 7), *k* = 12 (*n* = 7, *m* = 10), and *k* → ∞, respectively. **g**–**j** First-generation type IB tilings with $k = 6\, (\tilde n = 2,\tilde m = 1)$, $k = 9\, (\tilde n = 3,\tilde m = 2)$, $k = 12\, (\tilde n = 4,\tilde m = 3)$, and *k* → ∞, respectively. In **f**, **j** the *k* → ∞ patterns are drawn schematically, showing only a patch of majority tiles. **k**, **l** Subdivision patterns for the rectangle (R), small triangle (ST), and large triangle (LT) tiles in *k* = 6 type IA and IB tilings, respectively, which also illustrate the meaning of $n,m,\tilde n$, and $\tilde m$. **m** Second-generation *k* = 6 type IA tiling, with dark blue outlines showing the superimposed first-generation tiling magnified by a factor of $\beta _6 = 3 + \sqrt {10}$ so as to emphasize self-similarity

Here we complement the established concept of periodic approximants by introducing structures that locally resemble periodic crystals but are globally quasicrystalline (Fig. 1a). We focus on a class of aperiodic approximants that are characterized by hexagonal symmetry^23^ and originate in the bronze-mean tiling^24^ (Fig. 1b), their inflation factors being metallic means of multiples of 3 (Fig. 1c–j). By elaborating the subdivision rules, the higher-dimensional analysis, and the diffraction patterns of the aperiodic approximants as well as by discussing their non-metallic-mean variants, we provide a comprehensive description of this particular type of incommensurately modulated structures. Our findings offer a new perspective of the manifestation of six-fold symmetry, which may be very elaborate as demonstrated, e.g., by the binary nanoparticle assemblies featuring two-lengthscale in-plane local motifs identical to those reported here^25^ and by the twin-boundary superstructures theoretically predicted in monodisperse particles with a fairly simple pair interactions^26,27^.

## Results

### Quasicrystalline hexagonal tilings

We construct several types of two-lengthscale tilings with 6-fold symmetry, both composed of small (ST) and large (LT) equilateral triangles of edge length *S* and *L*, respectively, and of *L* × *S* rectangles (R) seen in the fundamental dodecagon of the bronze-mean tiling (Fig. 1b). In type IA tilings, the fundamental motif consist of a central rosette of 6 LT tiles, 6 radial spokes each containing *n* R tiles, and 6 wedges of ST tiles filling the gaps between the spokes (Fig. 1c–e and Supplementary Note 1). These tilings are additionally described by the number of rectangles along the short edge of the next-generation R tile denoted by *m* (Fig. 1k), or, equivalently, by the number of rows of ST tiles in the wedges of the fundamental motif measured in the radial direction as shown in Supplementary Fig. 2. Evidently *m* must be no smaller than *n*; for *m* = 2*n* the ST wedges reduce to rhombi.

As is common in self-similar patterns, the next-generation tiling can be obtained by magnifying the previous-generation tiling by a suitable factor and then placing the fundamental motifs at its vertices. Within the ST wedges of the next-generation tiling, the fundamental motifs partly overlap so as to create a gap-free pattern. On the other hand, the fundamental motifs fill out most but not all of the area of the previous-generation LT and R tiles.

Using this procedure, we find the subdivision scheme illustrated in Fig. 1k using the *n* = 3, *m* = 4 pattern. Upon subdivision, the two lengths of the *i*-th generation tiling *L*_*i*_ and *S*_*i*_ transform as

1 $$\left( {\begin{array}{*{20}{c}} {L_{i + 1}} \\ {S_{i + 1}} \end{array}} \right) = \left( {\begin{array}{*{20}{c}} 2 & {\sqrt 3 n} \\ {\sqrt 3 } & m \end{array}} \right)\left( {\begin{array}{*{20}{c}} {L_i} \\ {S_i} \end{array}} \right).$$

The positive eigenvalue of the transformation matrix given by

2 $$\lambda _ + ^{{\mathrm{IA}}} = \frac{{m + 2 + \sqrt {(m - 2)^2 + 12n} }}{2}$$

is the inflation factor and the corresponding eigenvector gives the length ratio at which the pattern is self-similar: $\phi _{{\mathrm{IA}}} = L/S = \left[ { - m + 2 + \sqrt {(m - 2)^2 + 12n} } \right]/(2\sqrt 3 ).$ Also of interest are the numbers of long and short edges $n_i^L$ and $n_i^S$, respectively, which transform according to

3 $$\left( {\begin{array}{*{20}{c}} {n_{i + 1}^L} \\ {n_{i + 1}^S} \end{array}} \right) = \left( {\begin{array}{*{20}{c}} 2 & {\sqrt 3 } \\ {\sqrt 3 n} & m \end{array}} \right)\left( {\begin{array}{*{20}{c}} {n_i^L} \\ {n_i^S} \end{array}} \right).$$

In a self-similar tiling, the ratio of long and short edges *ψ*_IA_ = *n*^*L*^/*n*^*S*^ is *ϕ*_IA_/*n*.

If *n* = 2*k*/3 − 1 and *m* = *k* − 2, then the inflation factor of this tiling equals the metallic mean

4 $$\beta _k = \frac{{k + \sqrt {k^2 + 4} }}{2}$$

(defined as the positive solution of the quadratic equation *x*^2^ − *kx* − 1 = 0), where *k* is a multiple of 3. By locking *n* and *m* with these two relations, we obtain a subset of type IA patterns which is of particular interest due to the singular role of metallic means in QCs epitomized by the golden and the silver mean behind the Penrose^28,29^ and the Ammann–Beenker tiling^30,31^, respectively. In the metallic-mean type IA tilings, the length ratio reads

5 $$\phi _{{\mathrm{IA}}} = \frac{{ - k + 4 + \sqrt {k^2 + 4} }}{{2\sqrt 3 }}$$

and the ratio of long and short edges is

6 $$\psi _{{\mathrm{IA}}} = \frac{{\sqrt 3 \left( { - k + 4 + \sqrt {k^2 + 4} } \right)}}{{2(2k - 3)}}.$$

In the bronze-mean *k* = 3 tiling, we have *ϕ* = *ψ*; for all other *k*s these ratios are different. Figure 1m shows the second-generation *k* = 6 type IA tiling, where $\phi _{{\mathrm{IA}}} = \left( { - 1 + \sqrt {10} } \right)/\sqrt 3 \approx 1.248$. Three more second-generation tilings are presented in Supplementary Note 2 where we also show zoomed-in portions of second- and third-generation *k* = 9 type IA and IB tilings, respectively, which provide an insight complementary to the complete flower-like patterns.

Type IA tiling is not the only 6-fold pattern that can be constructed using ST, LT, and R tiles. In type IB, the central LT rosette of the bronze-mean fundamental motif is enlarged so as to obtain a hexagonal patch of LT tiles with $\tilde n$ triangles along an edge (Fig. 1g–i). This patch is encompassed by a ring consisting of ST and R tiles. The outermost layer contains 6 spokes, each with $\tilde m$ radially oriented R tiles, and trapezoidal patches of LT tiles filling the space between the spokes. Like in type IA tilings, $\tilde m$ is chosen so as to maximize the size of the fundamental motif consistent with the construction of the next-generation tiling.

The analysis of inflation in type IB tilings is analogous to that in type IA tilings. The transformation matrix in type IB counterpart of Eq. (1) reads

7 $$\left( {\begin{array}{*{20}{c}} {2\tilde n + \tilde m} & {\sqrt 3 } \\ {\sqrt 3 \tilde n} & 1 \end{array}} \right)$$

and the matrix in the type IB version of Eq. (3) is its transpose. Like in type IA tilings, type IB inflation factors given by $\left[ {2\tilde n + \tilde m + 1 + \sqrt {(\tilde m - 1)^2 + 4\tilde n(2 + \tilde m + \tilde n)} } \right]/2$ reduce to metallic means *β*_*k*_ for $\tilde n = k/3$ and $\tilde m = k/3 - 1$ if *k* is a multiple of 3. In a general two-parameter type IB tiling, the self-similar length ratio and the ratio of long and short edges are given by $\phi _{{\mathrm{IB}}} = \left[ {2\tilde n + \tilde m - 1 + \sqrt {(\tilde m - 1)^2 + 4\tilde n(2 + \tilde m + \tilde n)} } \right]/(2\sqrt 3 \tilde n),$ and $\psi _{{\mathrm{IB}}} = \tilde n\phi _{{\mathrm{IB}}}$, respectively, which in the multiple-of-3 metallic-mean type IB tiling read $\phi _{{\mathrm{IB}}} = \sqrt 3 \left( {k - 2 + \sqrt {k^2 + 4} } \right)/(2k)$ and *ψ*_IB_ = *kϕ*_IB_/3, respectively.

The most important feature of type IA and type IB patterns is that as *k* is increased, they are dominated by ST and LT tiles, respectively, so that they approach a periodic hexagonal crystal (Fig. 1c–j). These two types are not the only possible self-similar metallic-mean tilings based on ST, LT, and R tiles. For each *k*, there exists a finite number of distinct types, each with a different transformation matrix and thus a different length ratio (Methods); the three additional *k* = 6 type I tilings are presented in Supplementary Note 3.

### Higher-dimensional representation

To provide a theoretical basis for these tilings, we construct their higher-dimensional description based on a 4-D superspace lattice with two lattice constants *a* and *c* rather than with a single one; in this respect, our analysis departs from existing constructions. We note that a generalization of the 6-D superspace representation of the bronze-mean hexagonal quasicrystal from ref. ^24^ is completely equivalent to the 4-D representation proposed below.

We denote the orthogonal basis vectors that generate the four-dimensional hyperspace by **e**_1_, **e**_2_, **e**_3_, and **e**_4_, where **e**_*i*_ ⋅ **e**_*j*_ = *δ*_*ij*_ and *δ*_*ij*_ is the Kronecker delta. The points of the superspace lattice are given by ${\mathbf{r}} = \mathop {\sum}\limits_{j = 1}^4 {n_j} {\mathbf{a}}_j$, where *n*_*j*_ are integers and the basis vectors **a**_*j*_ read *a* **u**_*j*_ for odd *j* and by *c* **u**_*j*_ for even *j*; **u**_*j*_ constitute a direct sum of two hexagonal bases {**u**_1_, **u**_3_} ⊕ {**u**_2_, **u**_4_}:

8 $${\mathbf{u}}_1 = \left( {\begin{array}{*{20}{c}} 1 \\ 0 \\ 0 \\ 0 \end{array}} \right),\:{\mathbf{u}}_2 = \left( {\begin{array}{*{20}{c}} 0 \\ 0 \\ 1 \\ 0 \end{array}} \right),\:{\mathbf{u}}_3 = \left( {\begin{array}{*{20}{c}} {1/2} \\ {\sqrt 3 /2} \\ 0 \\ 0 \end{array}} \right),\quad {\mathrm{and}}\quad {\mathbf{u}}_4 = \left( {\begin{array}{*{20}{c}} 0 \\ 0 \\ {1/2} \\ {\sqrt 3 /2} \end{array}} \right).$$

Suppose that the projected lattice allows a 30° rotation and scaling transformations: $\ell \to 1 \to \ell \to 1 \to \cdots$; here $\ell$ is a yet undetermined parameter. The transformation matrix is given by

9 $$T = \left( {\begin{array}{*{20}{c}} 0 & 0 & {\ell /2} & { - \sqrt 3 \ell /2} \\ 0 & 0 & {\sqrt 3 \ell /2} & {\ell /2} \\ {1/\ell } & 0 & 0 & 0 \\ 0 & {1/\ell } & 0 & 0 \end{array}} \right).$$

The real and imaginary parts of the eigenvector associated with the eigenvalue *z* = exp(*iπ*/6) of the *T* matrix are ${\mathbf{p}}_x^\parallel = \alpha \left( {1,0,\sqrt 3 \ell /2, - \ell /2} \right)$ and ${\mathbf{p}}_y^\parallel = \alpha \left( {0,1,\ell /2,\sqrt 3 \ell /2} \right)$, where $\alpha = 1/\sqrt {\ell ^2 + 1} .$ These two vectors span an invariant subspace called the physical space. Similarly, the complementary invariant subspace referred to as the perpendicular space is spanned by ${\mathbf{p}}_x^ \bot = \alpha \left( {\ell ,0, - \sqrt 3 /2,1/2} \right)$ and ${\mathbf{p}}_y^ \bot = \alpha \left( {0, - \ell ,1/2,\sqrt 3 /2} \right)$.

The projection operator onto the physical space is defined by $P_{jk}^\parallel = \mathop {\sum}\limits_{\gamma = x,{\kern 1pt} y} \; \left( {{\mathbf{p}}_\gamma ^\parallel \cdot {\mathbf{e}}_j} \right)\left( {{\mathbf{p}}_\gamma ^\parallel \cdot {\mathbf{e}}_k} \right)$ and reads

10 $$P^\parallel = \frac{1}{{\ell ^2 + 1}}\left( {\begin{array}{*{20}{c}} 1 & 0 & {\sqrt 3 \ell /2} & { - \ell /2} \\ 0 & 1 & {\ell /2} & {\sqrt 3 \ell /2} \\ {\sqrt 3 \ell /2} & {\ell /2} & {\ell ^2} & 0 \\ { - \ell /2} & {\sqrt 3 \ell /2} & 0 & {\ell ^2} \end{array}} \right).$$

Note that *P*^∥2^ = *P*^∥^. The complementary projection operator introduced by *P*^⊥^ = 1 − *P*^∥^ or, equivalently, by $P_{jk}^ \bot = \mathop {\sum}\limits_{\gamma = x,{\kern 1pt} y} \; \left( {{\mathbf{p}}_\gamma ^ \bot \cdot {\mathbf{e}}_j} \right)\left( {{\mathbf{p}}_\gamma ^ \bot \cdot {\mathbf{e}}_k} \right)$ is

11 $$P^ \bot = \frac{1}{{\ell ^2 + 1}}\left( {\begin{array}{*{20}{c}} {\ell ^2} & 0 & { - \sqrt 3 \ell /2} & {\ell /2} \\ 0 & {\ell ^2} & { - \ell /2} & { - \sqrt 3 \ell /2} \\ { - \sqrt 3 \ell /2} & { - \ell /2} & 1 & 0 \\ {\ell /2} & { - \sqrt 3 \ell /2} & 0 & 1 \end{array}} \right).$$

**Fig. 2:**
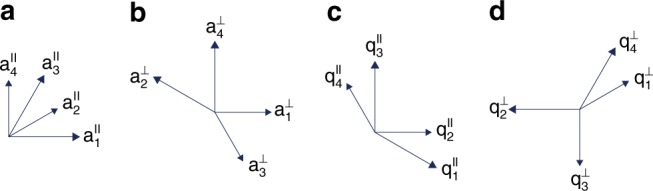
Basis vectors in real and in reciprocal space. **a**, **b** Projected basis vectors of *k* = 3 tilings in the physical and in the perpendicular space ${\mathbf{a}}_j^\parallel$ and ${\mathbf{a}}_j^ \bot$, respectively. **c**, **d** Projected reciprocal-space basis vectors in the physical and in the perpendicular space ${\mathbf{q}}_j^\parallel$ and ${\mathbf{q}}_j^ \bot$, respectively

Using *P*^∥^ and *P*^⊥^, we define the basis vectors in the physical and in the perpendicular space by ${\mathbf{a}}_j^\parallel \equiv P^\parallel {\mathbf{a}}_j$ and ${\mathbf{a}}_j^ \bot \equiv P^ \bot {\mathbf{a}}_j$, respectively. Thus we find that $\left| {{\mathbf{a}}_{{\mathrm{odd}}}^\parallel } \right| = a{\mkern 1mu} \ell {\mkern 1mu} \alpha$, $\left| {{\mathbf{a}}_{{\mathrm{even}}}^\parallel } \right| = c{\mkern 1mu} \alpha$, $\left| {{\mathbf{a}}_{{\mathrm{odd}}}^ \bot } \right| = a{\mkern 1mu} \alpha$, and $\left| {{\mathbf{a}}_{{\mathrm{even}}}^ \bot } \right| = c{\mkern 1mu} \ell {\mkern 1mu} \alpha$. The ratios of the lengths of even and odd basis vectors in the physical and in the perpendicular space are

12 $$\frac{{\left| {{\mathbf{a}}_{{\mathrm{odd}}}^\parallel } \right|}}{{\left| {{\mathbf{a}}_{{\mathrm{even}}}^\parallel } \right|}} = \frac{{a\ell }}{c}\quad {\mathrm{and}}\quad \frac{{\left| {{\mathbf{a}}_{{\mathrm{even}}}^ \bot } \right|}}{{\left| {{\mathbf{a}}_{{\mathrm{odd}}}^ \bot } \right|}} = \frac{{c\ell }}{a},$$

respectively. The *x* and *y* components of ${\mathbf{a}}_j^\parallel$ in the physical space and those of ${\mathbf{a}}_j^ \bot$ in the perpendicular space are given by $\left( {{\mathbf{a}}_j^\parallel \cdot {\mathbf{p}}_x^\parallel ,{\mathbf{a}}_j^\parallel \cdot {\mathbf{p}}_y^\parallel } \right)$ and $\left( {{\mathbf{a}}_j^ \bot \cdot {\mathbf{p}}_x^ \bot ,{\mathbf{a}}_j^ \bot \cdot {\mathbf{p}}_y^ \bot } \right),$ respectively, and the projected basis vectors of *k* = 3 tilings are shown in Fig. 2. The basis vectors for *k* = 6, 9, 12, and ∞ type IA and IB tilings are discussed in Supplementary Note 4.

Now we need to choose the two parameters $\ell$ and *c*/*a* so as to obtain a desired metallic-mean hexagonal QC. The simplest way of introducing the characteristic lengthscale ratio in the physical space and ensuring that the projection window is compact is to require that $\left| {{\mathbf{a}}_{{\mathrm{odd}}}^\parallel } \right|/\left| {{\mathbf{a}}_{{\mathrm{even}}}^\parallel } \right| = \phi _{{\mathrm{IA}}}$ and to choose $\left| {{\mathbf{a}}_{{\mathrm{even}}}^ \bot } \right|/\left| {{\mathbf{a}}_{{\mathrm{odd}}}^ \bot } \right| = \psi _{{\mathrm{IA}}}$ for the type IA tiling [where *ϕ*_IA_ and *ψ*_IA_ are given by Eqs. (5) and (6), respectively] and analogously for the other type I tilings. These conditions imply that for type IA tiling

13 $$\left( {\frac{c}{a}} \right)_{{\mathrm{IA}}} = \sqrt {\frac{{2k - 3}}{3}}$$

and

14 $$\ell _{{\mathrm{IA}}} = \frac{{ - k + 4 + \sqrt {k^2 + 4} }}{{2\sqrt {2k - 3} }}.$$

In type IB tiling, $\left( {c/a} \right)_{{\mathrm{IB}}} = \sqrt {3/k}$ and $\ell _{{\mathrm{IB}}} = \left( {\sqrt {k^2 + 4} + k - 2} \right)/(2\sqrt k ),$ respectively. The values of these parameters for *k* = 3, 6, 9, and 12 as well as the corresponding ratios of basis vectors for type IA and IB tilings are tabulated in Supplementary Tables 2 and 3. Evidently the ratio of superspace lattice constants *c*/*a* is different from unity in all of our tilings except in the bronze-mean *k* = 3 QCs.

**Fig. 3:**
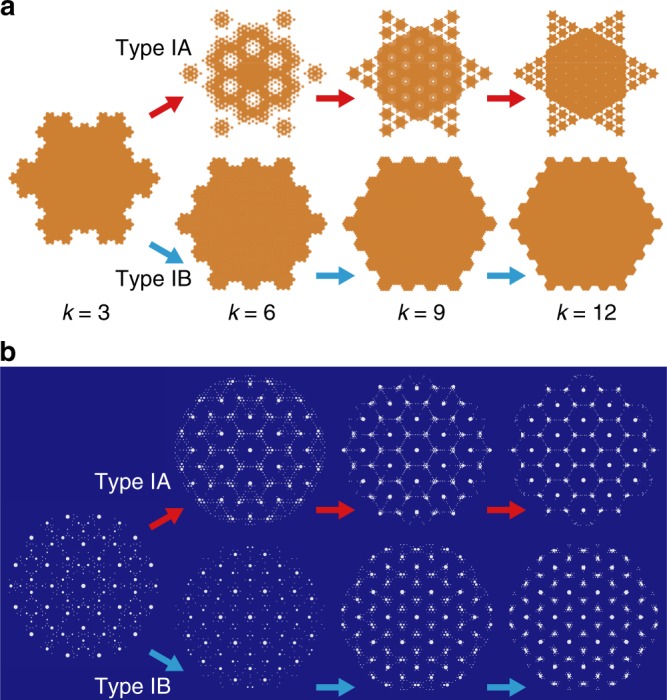
Projection windows and Fourier transforms. **a** Windows of the *k* = 3, 6, 9, and 12 type IA tilings with 324391, 123392, 1212652, and 7195910 points, respectively (top row), and type IB tilings with 95659, 11041, and 32767 points, respectively; the windows are obtained based on third-generation tilings except for the *k* = 3 tiling (fifth generation) and *k* = 12 type IB tiling (second generation). **b** Transforms of these tilings with spots normalized relative to the central peak; shown are all spots with intensities *I*(**q**^∥^) > 0.0045

Using these ratios, we now construct the projection windows in the perpendicular space. The position of a vertex of a tiling in the physical space is described by ${\mathbf{r}}^\parallel = \mathop {\sum}\limits_{j = 1}^4 {n_j} {\mathbf{a}}_j^\parallel$, where *n*_*j*_ are integers and ${\mathbf{a}}_j^\parallel$ are the physical-space basis vectors of the tiling; inflation of tilings is elaborated in Methods. The same set of *n*_*i*_ also defines the position of the corresponding vertex in the perpendicular space ${\mathbf{r}}^ \bot = \mathop {\sum}\limits_{j = 1}^4 {n_j} {\mathbf{a}}_j^ \bot$, and these vertices constitute the projection windows. Figure 3a shows the projection windows for the *k* = 3, 6, 9, and 12 type IA and type IB tilings from Fig. 1b–e, g–i, which evidently tend to self-similar shapes as one proceeds with inflation from generation to generation.

Apart from their physical-space structure, the key signature of each of our metallic-mean tilings is its Fourier transform which captures the main features of its diffraction image (Methods). Figure 3b shows the transforms of finite but large type IA and type IB tilings with *k* = 3, 6, 9, and 12, with the intensities normalized by the central peak; the transforms of the *k* = 6 tilings are discussed in more detail in Supplementary Note 5. As *k* is increased the image clearly approaches the pattern characteristic for the hexagonal lattice, with the incommensurate peaks becoming ever smaller and ever closer to those representing the hexagonal lattice. The strongest peaks in *k* = 6, 9, and 12 type IA patterns have reciprocal-space vectors with indices (2, 1, 0, −1), whereas the strongest peaks in *k* = 6, 9, and 12 type IB have reciprocal-space vectors with indices (2, 1, 1, 0).

The hierarchical nature of the transforms with the prominent peaks and many smaller ones is rather evident, and the weaker peaks are consistent with the satellite reflections known from samples with a periodic arrangement of (anti)phase-domain boundaries^26,32^, which gives rise to an incommensurately modulated periodic crystal if the period of the boundaries is not commensurate with that of the arrangement of atoms or other building blocks as appropriate^8^. With their characteristic structure consisting of domains forming a quasiperiodic pattern, our metallic-mean tilings can indeed be regarded as a special class of incommensurately modulated structures with an underlying periodic crystal and a set of modulation vectors from a quasicrystal with 6-fold symmetry.

### Hexagonal lattice as *k* → ∞ limit

The first-generation type IA and type IB sequences in Fig. 1b–j show that at large *k*s, these tilings are dominated by ST and LT tiles, respectively. By inspecting the patterns in Fig. 1k, we find that in type IA tiling the numbers of ST, LT, and R tiles in the (*i* + 1)-th generation denoted by *ST*_*i*+1_, *LT*_*i*+1_, and *R*_*i*+1_, respectively, are related to those in the *i*-th generation by

15 $$\left( {\begin{array}{*{20}{c}} {ST_{i + 1}} \\ {LT_{i + 1}} \\ {R_{i + 1}} \end{array}} \right) = \left( {\begin{array}{*{20}{c}} {(k - 2)^2} & {\frac{{(3 - 2k)^2}}{3}} & {\frac{{4(2k^2 - 7k + 6)}}{3}} \\ 3 & 4 & 8 \\ {\frac{{3(k - 2)}}{2}} & {2k - 3} & {4k - 7} \end{array}} \right)\left( {\begin{array}{*{20}{c}} {ST_i} \\ {LT_i} \\ {R_i} \end{array}} \right).$$

**Fig. 4:**
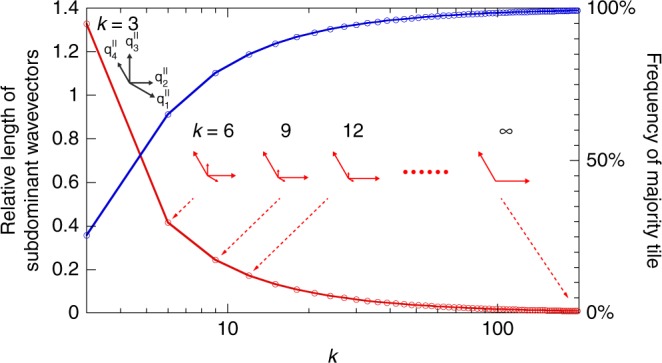
Convergence towards periodic hexagonal lattice. Ratio of magnitudes of subdominant and dominant reciprocal-space basis vectors (red points connected so as to guide the eye) and the frequencies of majority tiles in type IA tilings (blue points) vs. *k*. Also included are the reciprocal-space basis vectors for *k* = 3, 6, 9, 12, and ∞

The largest eigenvalue of the above matrix is $\beta _k^2$. Its top-row components scale as *k*^2^ for large *k*s whereas the middle- and the bottom-row components scale as 1 and *k*, respectively. As a result, ST tiles quickly outnumber LT and R tiles upon inflation, their frequency approaching unity at large *k*s (Fig. 4; Methods). An analogous argument shows that in large-*k* type IB tilings the majority tiles are LTs, further proving that the *k* → ∞ limit of both types is the periodic hexagonal lattice. Convergence towards the hexagonal lattice is also reflected in the fact that although the rank of the Fourier module is 4 for all *k*, 2 of the wave vectors become vanishingly small as *k* → ∞ and so does the intensity of the corresponding incommensurate peaks. In this limit, only two reciprocal-space wavevectors remain finite (${\mathbf{q}}_2^\parallel$ and ${\mathbf{q}}_4^\parallel$ in type IA tilings, ${\mathbf{q}}_1^\parallel$ and ${\mathbf{q}}_3^\parallel$ in type IB tilings, etc.) as implied by Fig. 3b and illustrated in the inset in Fig. 4 and discussed in Supplementary Note 4. This, in turn, means that for large *k*, the relative intensity of the spots *I*(**q**^∥^)/*I*(**0**) = 1.0 for any **q**^∥^ which is characteristic of the periodic hexagonal lattice. Yet another indication of the convergence is provided by the four basis vectors of the physical space, which cease to be linearly independent as *k* → ∞ (Supplementary Note 4).

### Complementary tilings and accidental periodic crystals

**Fig. 5:**
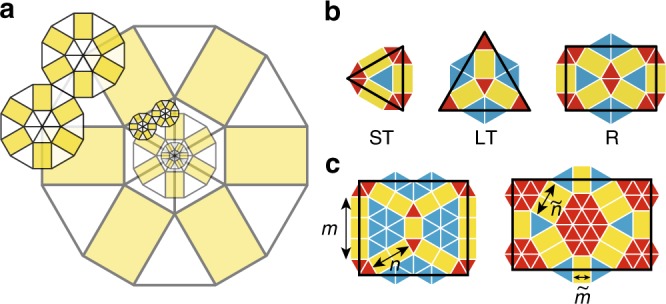
Type II tilings. **a** Dodecagonal type IIA fundamental motif combined with four second-generation dodecagons and three third-generation dodecagon. Only the R tiles are colored so as to emphasize rotation by 30° upon subdivision; some are semitransparent for clarity. **b** Type IIA subdivision scheme. **c** Subdivision scheme for generalized type IIA and type IIB R tiles parametrized by (*m*, *n*) and $(\tilde m,\tilde n)$, respectively

The different type I tilings hardly exhaust the possible structures formed by ST, LT, and R tiles. In a variant of the bronze-tiling mentioned in passing in ref. ^24^ and observed in ref. ^25^, the fundamental motif consists of six ST tiles arranged in a hexagonal rosette (Fig. 1k of ref. ^24^), six R tiles around them pointing in the radial direction, and six LT tiles filling the gaps between the R tiles. Upon inflation, this motif becomes decorated by smaller copies of itself rotated by 30° relative to the first-generation tiling; the orientation of the third-generation dodecagons is again the same as in the first generation (Fig. 5a). In this respect, this pattern referred to as type II is quite different from type I tilings.

Like in type I tilings, we can construct generalized subdivision schemes for type II tilings parametrized by *n* and *m* (type IIA) and $\tilde n$ and $\tilde m$ (type IIB) as illustrated by the R tiles in Fig. 5c. In type IIA, we have

16 $$\left( {\begin{array}{*{20}{c}} {L_{i + 1}} \\ {S_{i + 1}} \end{array}} \right) = \left( {\begin{array}{*{20}{c}} {\sqrt 3 n} & 2 \\ m & {\sqrt 3 } \end{array}} \right)\left( {\begin{array}{*{20}{c}} {L_i} \\ {S_i} \end{array}} \right).$$

The columns in this transformation matrix are swapped compared to the matrix for type IA tilings [Eq. (1)]. Like in type I tilings, this matrix gives the inflation factor and the self-similar length ratio which read $\left[ {\sqrt 3 (n + 1) + \sqrt {3(n - 1)^2 + 8m} } \right]/2$ and $\left[ {\sqrt 3 (n - 1) + \sqrt {3(n - 1)^2 + 8m} } \right]/(2m)$, respectively. In the *n* = *m* = 1 case shown in Fig. 5a, b, the inflation factor is $\sqrt 2 + \sqrt 3$ which is a non-Pisot number^33^ but its square $5 + 2\sqrt 6$ corresponding to two consecutive subdivisions is; its self-similar length ratio is $\sqrt 2$. Without going into details, we note that type IIB tiling is related to type IIA tiling just as type IB is related to type IA, and that the meaning of $\tilde n$ and $\tilde m$ is analogous as illustrated in Fig. 5c.

Although the main emphasis of the above discussion was on the multiple-of-3 metallic-mean patterns, type II tilings show that the general two-parameter tiling scheme based on ST, LT, and R tiles is very rich, containing an infinite class of self-similar tilings of 6-fold rotational symmetry concisely represented by their inflation factors. In Table 1 we list the inflation factors of type IA tilings given by Eq. (2) for *n* between 1 and 5 and *m* between *n* and *n* + 6, which emphasizes that the multiple-of-3 metallic-mean tilings are just a small subset of the two-parameter ST/LT/R scheme; those for type IB and type IIA tilings are discussed in Supplementary Note 6 and listed in Supplementary Tables 4 and 5. Among the interesting non-metallic-mean special cases, we single out the *n* = 1, *m* = 2 type IA square-triangle pattern with the inflation factor of $2 + \sqrt 3$ known from the usual dodecagonal QCs, whose hexagonal arrangement was already studied^33–36^. Also intriguing are the generalized type IA and IB tilings with *m* = 3*n*/2 and $\tilde m = \tilde n$, respectively, which have integer inflation factors (Table 1 and Supplementary Table 4). As the points in their diffraction pattern are placed at those of the triangular lattice but are not dense, the type IA tilings with *m* = 3*n*/2 are limit-periodic rather than aperiodic^33,37^. On the other hand, the $\tilde n = \tilde m$ type IB tilings which too have integer inflation factors are periodic crystalline rather than quasicrystalline because they consist of a single type of domains as shown by Supplementary Fig. 12. Given that these tilings are still characterized by a single inflation factor (which is equal to $3\tilde n + 1$) their periodicity appears to be accidental. Together with the main body of results presented above, these examples illustrate how our hexagonal QCs contribute to the generic theory of tilings, especially to the *k*-uniform tilings that are not based on regular polygons^30^.

**Table 1:** Inflation factors of type IA tilings with 1 ≤ *n* ≤ 5 and *n* ≤ *m* ≤ *n* + 6^a^

*m*	*n*				
	1	2	3	4	5
*n*	$\frac{{{\mathbf{3}} + \sqrt {{\mathbf{13}}} }}{{\mathbf{2}}}$	$2 + \sqrt 6$	$\frac{{5 + \sqrt {37} }}{2}$	$3 + \sqrt {13}$	$\frac{{7 + \sqrt {69} }}{2}$
*n* + 1	$2 + \sqrt 3$	5	${\mathbf{3 + }}\sqrt {{\mathbf{10}}}$	$\frac{{7 + \sqrt {57} }}{2}$	$4 + \sqrt {19}$
*n* + 2	$\frac{{5 + \sqrt {13} }}{2}$	$3 + \sqrt 7$	$\frac{{7 + 3\sqrt 5 }}{2}$	8	$\frac{{{\mathbf{9 + }}\sqrt {{\mathbf{85}}} }}{{\mathbf{2}}}$
*n* + 3	5	$\frac{{7 + \sqrt {33} }}{2}$	$4 + \sqrt {13}$	$\frac{{9 + \sqrt {73} }}{2}$	$5 + 2\sqrt 6$
*n* + 4	$\frac{{7 + \sqrt {21} }}{2}$	$4 + \sqrt {10}$	$\frac{{9 + \sqrt {61} }}{2}$	$5 + \sqrt {21}$	$\frac{{11 + \sqrt {109} }}{2}$
*n* + 5	$4 + \sqrt 7$	8	$5 + 3\sqrt 2$	$\frac{{11 + \sqrt {97} }}{2}$	$6 + \sqrt {31}$
*n* + 6	$\frac{{9 + \sqrt {37} }}{2}$	$5 + \sqrt {15}$	$\frac{{11 + \sqrt {85} }}{2}$	$2(3 + \sqrt 7 )$	$\frac{{13 + \sqrt {141} }}{2}$

^a^The multiple-of-3 metallic-mean inflation factors are typeset in boldface

## Discussion

The hexagonal QCs based on ST, LT, and R tiles can be directly related to two-lengthscale superstructures seen in several systems including binary nanoparticles^25^, intermetallic alloys^38^, and oxide QC approximants^39^, which do contain such tiles. These experimentally observed structures are generally less neatly ordered than, e.g., the pattern in Fig. 1m, which may be attributed to phason flips^40^ whereby the periodic domains can either expand or shrink by collective displacement of a row of R tiles forming the twin boundary. The ensuing structures can thus be regarded as randomized versions of our QCs^41,42^. It is natural to wonder what may be the possible physical mechanisms behind the generally large distance between the domain/twin boundaries and the sophisticated patterns such as that in Fig. 1m. The theoretical insight into domain-wall incommensurate phases in systems with a simple isotropic pair interaction^26,27^ suggests that the forces needed to stabilize such patterns need not be very complex, implying that they may well appear in an atomic or colloidal material even if it consists of a single type of particles.

The late members of the multiple-of-3 metallic-mean self-similar hexagonal QCs described here approach the periodic hexagonal crystal; the non-metallic-mean QCs consisting of small and large triangles and rectangles must do so too. As such, these QCs can be regarded as aperiodic approximants of periodic crystals formed by increasing the size of their domains that consist of majority triangles, much like periodic approximants approach QCs as the size of their unit cells grows. We expect that there exist other types of aperiodic approximants with traditionally allowed rotational symmetry. For example, it is conceivable that 4-fold approximants may be derived from the Ammann–Beenker tiling by replacing the single-length square-rhombus tiling scheme by a two-lengthscale set of tiles including a small and large square and a parallelogram. Whether our approach can be used to construct approximants of QCs with a rotational symmetry that is coprime to any periodic crystal (e.g., 5-fold) remains an open question.

The aperiodic approximants proposed here resonate with two established concepts in QCs. Firstly, our hexagonal QCs can be likened to the random square-triangle tilings^41–43^ which constitute the dodecagonal QC if the triangle-to-square ratio is $4/\sqrt 3 \approx 2.3094$, and reduce to the hexagonal and the square lattice if the ratio is infinite and 0, respectively. At large but finite ratios, these tilings consist of random hexagonal-lattice domains separated by domain walls of squares; our QCs are domain structures too and they approach the hexagonal lattice in the limit of *k* → ∞ but the domains form a regular hierarchical pattern. At the same time, we can draw an analogy between our aperiodic approximants and the well-known 1-D Fibonacci QCs^44^ based on the substitution rule A → A^*k*^B, B → A. These QCs can too be viewed as a simple type of sequence of aperiodic approximants because as *k* → ∞, the number ratio of the short and the long segments vanishes so that the Fibonacci QCs approach a 1-D periodic crystal. Together with the experimental indications mentioned above, these parallels support the notion of apeiodic approximants as incommensurately modulated structures characterized by locally periodic but globally quasiperiodic positional order.

## Methods

### Generalized transformation matrix and distinct types of tilings

Type IA and IB tilings do not exhaust the metallic-mean patterns at a given *k*; at *k* = 6, there exist a total of five of them shown in Supplementary Fig. 8. To see how the different types emerge, note that the matrices describing the transformation of the long and the short length of type IA and type IB patterns [Eqs. (1) and (7)] can be viewed as special cases of a generalized transformation matrix of the form

17 $$\left( {\begin{array}{*{20}{c}} {L_{i + 1}} \\ {S_{i + 1}} \end{array}} \right) = \left( {\begin{array}{*{20}{c}} \alpha & {\sqrt 3 \beta } \\ {\sqrt 3 \gamma } & \delta \end{array}} \right)\left( {\begin{array}{*{20}{c}} {L_i} \\ {S_i} \end{array}} \right),$$

where *α*, *β*, *γ*, and *δ* are positive integers. If this matrix is to define a subdivision rule, *α* must be larger than 1. The characteristic equation of the matrix is *λ*^2^ − (*α* + *δ*)*λ* + *αδ* − 3*βγ* = 0. If *α* + *δ* = *k* and *αδ* − 3*βγ* = −1, then the positive eigenvalue of the matrix, which represents the inflation factor, is the metallic mean *β*_*k*_. This shows that there exist several combinations of *α*, *β*, *γ*, and *δ* that lead to the same metallic-mean eigenvalue.

For *k* = 3, the only choice of the four parameters consistent with the constraints is *α* = 2, *β* = 1, *γ* = 1, and *δ* = 1 but for *k* = 6 there are six combinations listed in Supplementary Table 1 and arranged in ascending order of *α* and *β*. Two of them represent type IA and type IB tilings, which are elaborated in the main text, and the remaining four are described in Supplementary Note 3. In Supplementary Table 1, we also list the self-similar length ratio *L*/*S* defined by the eigenvector corresponding to the positive eigenvalue of the transformation matrix in Eq. (17), which reads

18 $$\phi (\alpha ,\beta ,\gamma ,\delta ) = \frac{{\alpha - \delta + \sqrt {\alpha ^2 + 12\beta \gamma - 2\alpha \delta + \delta ^2} }}{{2\sqrt 3 \gamma }}.$$

The construction of type IC-F tilings proceeds by deriving the subdivision rules. Using the transformation matrix, we first determine the number of tiles with long and short edges to fit along either edge of the R tile as well as their orientation, the latter pertaining to ST and LT tiles. After that, we arrange the second-generation tiles along the four edges and then we fill out the rest of the R tile such that the subdivision pattern is characterized by two perpendicular mirror planes through the center and parallel to the edges. This involves some educated guessing as well as some trial and error. After the subdivision rule for the R tile is established, we also construct the rules for the LT and ST tiles, again starting from a given arrangement of second-generation tiles along the edges. This procedure leads to patterns presented in Supplementary Note 3.

### Inflation of tilings

Upon inflation, **r**^∥^ of the *i*-th generation tiling is transformed to ${\mathbf{r}}^\parallel = \mathop {\sum}\limits_{j = 1}^4 {n_j} (i + 1){\mathbf{a}}_j^\parallel$ in the (*i* + 1)-th generation tiling. The transformation matrix relating the tiling-vector indices of the *i*-th generation and the (*i* + 1)-st generation general type IA tiling parametrized by *n* and *m* is

19 $$\left( {\begin{array}{*{20}{c}} {n_1(i + 1)} \\ {n_2(i + 1)} \\ {n_3(i + 1)} \\ {n_4(i + 1)} \end{array}} \right) = \left( {\begin{array}{*{20}{c}} 2 & 1 & 0 & { - 1} \\ {2n} & m & n & 0 \\ 0 & 1 & 2 & 2 \\ { - n} & 0 & n & m \end{array}} \right)\left( {\begin{array}{*{20}{c}} {n_1(i)} \\ {n_2(i)} \\ {n_3(i)} \\ {n_4(i)} \end{array}} \right).$$

The transformation matrix relating the tiling-vector indices of the *i*-th generation and the (*i* + 1)-st generation general type IB tiling parametrized by $\tilde n$ and $\tilde m$ is

20 $$\left( {\begin{array}{*{20}{l}} {n_1(i + 1)} \hfill \\ {n_2(i + 1)} \hfill \\ {n_3(i + 1)} \hfill \\ {n_4(i + 1)} \hfill \end{array}} \right) = \left( {\begin{array}{*{20}{cccc}} {2\tilde n + \tilde m} \hfill & {\tilde n} & 0 & { - \tilde n} \hfill \\ 2 & 1 & 1 & 0 \\ 0 & {\tilde n} & {2\tilde n + \tilde m} & {2\tilde n} \\ { - 1} & 0 & 1 & 1 \end{array}} \right)\left( {\begin{array}{*{20}{l}} {n_1(i)} \\ {n_2(i)} \\ {n_3(i)} \\ {n_4(i)} \end{array}} \right).$$

### Inflation matrices for tiles

To compute the frequency of the majority tiles, we examine the numbers of ST, LT, and R tiles in type IA and type IB tilings; these numbers in the *i*-th generation tiling are denoted by *ST*_*i*_,*LT*_*i*_, and *R*_*i*_, respectively. We first spell out the recursive relation for a general type IA tiling parametrized by *n* and *m*

21 $$\left( {\begin{array}{*{20}{c}} {ST_{i + 1}} \\ {LT_{i + 1}} \\ {R_{i + 1}} \end{array}} \right) = \left( {\begin{array}{*{20}{c}} {m^2} & {3n^2} & {4nm} \\ 3 & 4 & 8 \\ {\frac{3}{2}m} & {3n} & {3n + 2m} \end{array}} \right)\left( {\begin{array}{*{20}{c}} {ST_i} \\ {LT_i} \\ {R_i} \end{array}} \right),$$

and for general type IB tiling parametrized by $\tilde n$ and $\tilde m$

22 $$\left( {\begin{array}{*{20}{c}} {ST_{i + 1}} \\ {LT_{i + 1}} \\ {R_{i + 1}} \end{array}} \right) = \left( {\begin{array}{*{20}{c}} 1 & 3 & 4 \\ {3\tilde n^2} & {\tilde m^2 + 4\tilde n{\mathrm{ }}\tilde m + 4{\mathrm{ }}\tilde n^2} & {8\tilde n^2 + 4\tilde m{\mathrm{ }}\tilde n} \\ {\frac{{3\tilde n}}{2}} & {\frac{{3(\tilde m + 2\tilde n)}}{2}} & {\tilde m + 5\tilde n} \end{array}} \right)\left( {\begin{array}{*{20}{c}} {ST_i} \\ {LT_i} \\ {R_i} \end{array}} \right).$$

In the metallic-mean type IA tilings where *n* = 2*k*/3 − 1 and *m* = *k* − 2,

23 $$\left( {\begin{array}{*{20}{c}} {ST_{i + 1}} \\ {LT_{i + 1}} \\ {R_{i + 1}} \end{array}} \right) = \left( {\begin{array}{*{20}{c}} {(k - 2)^2} & {\frac{{(3 - 2k)^2}}{3}} & {\frac{{4(k^2 - 7k + 6)}}{3}} \\ 3 & 4 & 8 \\ {\frac{{3(k - 2)}}{2}} & {2k - 3} & {4k - 7} \end{array}} \right)\left( {\begin{array}{*{20}{c}} {ST_i} \\ {LT_i} \\ {R_i} \end{array}} \right),$$

whereas in the metallic-mean type IB tilings where $\tilde n = k/3$ and $\tilde m = k/3 - 1$,

24 $$\left( {\begin{array}{*{20}{c}} {ST_{i + 1}} \\ {LT_{i + 1}} \\ {R_{i + 1}} \end{array}} \right) = \left( {\begin{array}{*{20}{c}} 1 & 3 & 4 \\ {\frac{{k^2}}{3}} & {(k - 1)^2} & {\frac{{4(k - 1)k}}{3}} \\ {\frac{k}{2}} & {\frac{{3(k - 1)}}{2}} & {2k - 1} \end{array}} \right)\left( {\begin{array}{*{20}{c}} {ST_i} \\ {LT_i} \\ {R_i} \end{array}} \right),$$

The largest eigenvalue of matrices in Eqs. (23) and (24) is $\beta _k^2 = \left( {k^2 + k\sqrt {k^2 + 4} + 2} \right)/2$. The first component of the corresponding eigenvector in type IA tilings is

25 $$\frac{{16k^2 - 15\sqrt {k^2 + 4} - 39k + 60}}{{2k(8k - 15) + 75}},$$

which amounts to 0.652, 0.787, and 0.849 for *k* = 6, 9, and 12, respectively. As *k* → ∞, type IA tiling is evidently dominated by ST tiles as the majority tile as shown in Fig. 4 of the main text, which implies that it reduces to the hexagonal lattice. In type IB tilings, the majority tiles in the limit of *k* → ∞ are LT tiles as witnessed by the relative magnitude of entries in the top, middle, and bottom row of the matrix in Eq. (24). Their frequency is given by the second component of the largest-eigenvalue eigenvector is

26 $$\frac{{k(3\sqrt {k^2 + 4} + 7k - 6) - 12(\sqrt {k^2 + 4} - 2)}}{{k(10k - 3) + 48}},$$

giving 0.713, 0.813, and 0.862 for *k* = 6, 9, and 12, respectively. This shows that as *k* → ∞, type IB tilings also converge to the hexagonal lattice.

### Fourier transform

To evaluate the Fourier transform of the metallic-mean tiling exactly, we first define the reciprocal-space basis vectors ***q***^∥^ such that **a**_*j*_ ⋅ **q**_*j*′_ = 2*πδ*_*jj*'_, where *δ*_*jj*'_ is the Kronecker delta: **q**_*j*_ are given by (2*π*/*a*)**v**_*j*_ for odd *j* and by (2*π*/*c*)**v**_*j*_ for even *j*, where

27 $${\mathbf{v}}_1 = \left( {\begin{array}{*{20}{c}} 1 \\ { - 1/\sqrt 3 } \\ 0 \\ 0 \end{array}} \right),\:{\mathbf{v}}_2 = \left( {\begin{array}{*{20}{c}} 0 \\ 0 \\ 1 \\ { - 1/\sqrt 3 } \end{array}} \right),\:{\mathbf{v}}_3 = \left( {\begin{array}{*{20}{c}} 0 \\ {2/\sqrt 3 } \\ 0 \\ 0 \end{array}} \right),\quad {\mathrm{and}}\quad {\mathbf{v}}_4 = \left( {\begin{array}{*{20}{c}} 0 \\ 0 \\ 0 \\ {2/\sqrt 3 } \end{array}} \right).$$

The projected reciprocal-space basis vectors are shown in Fig. 2c, d, respectively. The ratios of lengths of the basis vectors in the physical and the perpendicular space are $\left| {{\mathbf{q}}_{{\mathrm{odd}}}^\parallel } \right|/\left| {{\mathbf{q}}_{{\mathrm{even}}}^\parallel } \right| = c\ell /a = \psi$ and $\left| {{\mathbf{q}}_{{\mathrm{even}}}^ \bot } \right|/\left| {{\mathbf{q}}_{{\mathrm{odd}}}^ \bot } \right| = a\ell /c = \phi ,$ respectively. These ratios are the inverses of those in the physical space.

The following identity holds for any pair of vectors ***x***(*k*) = (***x***^∥^, ***x***^⊥^) and **q** = (**q**^∥^, **q**^⊥^): 1 = exp(*i***q** ⋅ **x**(*k*)) = exp(*i***q**^∥^ ⋅ **x**^∥^)exp(*i***q**^⊥^ ⋅ **x**^⊥^). If the particles’ positions are described by *δ*-functions so that the density reads $f({\mathbf{r}}^\parallel ) = \mathop {\sum}\limits_{k = 1}^N \delta \left( {{\mathbf{r}}^\parallel - {\mathbf{x}}^\parallel (k)} \right)$ where the sum goes over all particles, then the Fourier transform of the density is given by ${\int} d {\mathbf{r}}^\parallel \exp \left( { - i{\mathbf{q}}^\parallel \cdot {\mathbf{r}}^\parallel } \right)f({\mathbf{r}}^\parallel ) = \mathop {\sum}\limits_{k = 1}^N {\exp } \left( { - i{\mathbf{q}}^\parallel \cdot {\mathbf{x}}^\parallel (k)} \right) = \mathop {\sum}\limits_{k = 1}^N {\exp } \left( {i{\mathbf{q}}^ \bot \cdot {\mathbf{x}}^ \bot (k)} \right).$

## Supplementary information

Supplementary Information

## Data Availability

The whole datasets are available from the corresponding author on reasonable request.

## Code availability

The codes used to construct the tilings and the projection windows are available from the corresponding author on reasonable request.
