# Supplementary material for: Metallic-mean quasicrystals as aperiodic approximants of periodic crystals
Source: Nat Commun. 2019 Sep 17;10:4235. doi: 10.1038/s41467-019-12147-z (PMC6748959; doi:10.1038/s41467-019-12147-z)
Supplement: Supplementary file 1 — Supplementary Information [file 41467_2019_12147_MOESM1_ESM.pdf]

Metallic-mean quasicrystals as aperiodic approximants of periodic crystals

Nakakura et al.

Supplementary Note 1

Shape of fundamental motifs

Type IA tilings are parametrized by two integers defined by the subdivision patterns of the ST, LT, and R tile shown in Fig. 1k of the main text. To obtain the second-generation tiling from the first-generation tiling, we may either apply the subdivision rules in Fig. 1k or place the second-generation fundamental motifs at the vertices of the first-generation tiling. Generally, the thus placed second-generation fundamental motifs at the neighboring vertices partly overlap. If we choose the type IA fundamental motif such that its six LT domains are triangular as shown in Supplementary Fig. 1a, then the overlap of two such motifs along the short edges of the first-generation tiling and thus the m parameter of the subdivision rule in Fig. 1k of the main text are not unambiguously defined. In the sketch shown in Supplementary Fig. 1a, they overlap by 2 R tiles (so that $m = 4$) but they could also be pushed 1 R tile closer to each other (so that m would be 3) or 1 and 2 R tiles farther apart (so that m would be 5 and 6, respectively).

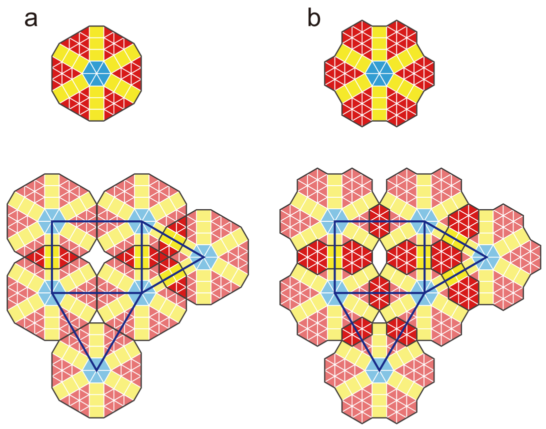

Supplementary Figure 1: Two choices for the fundamental motifs for $k = 6$ type IA tiling. Panel a shows a smaller motif which, when placed at the vertices of the ST, LT, and R tiles, does not define the value of m that characterizes the subdivision rule in Fig. 1k of the main text. The motif in panel b removes the ambiguity when packed along the short edges of the first-generation tiling such that the overlap is as large as possible. In bottom row, the fundamental motifs are semitransparent so as to highlight the overlap.

To fix the value of m, we add ST tiles at the outer edge of the triangular ST wedges (Supplementary Fig. 1b); in the case shown, we add a single row containing 5 ST tiles. These fundamental motifs are then packed such that along the short edges of the second-generation tiling the overlap is maximal, which defines m. The uncovered area in the centers of the first-generation R and LT tiles can be filled out unambiguously using second-generation tiles.

The thus defined fundamental motif offers a clear interpretation of the two parameters of type IA tiling, n and m: The former gives the number of R tiles in the 6 spokes radiating from the central rosette containing 6 LT tiles and the latter gives the number of rows in the diamond-shaped wedges containing ST tiles measured in the radial direction (Supplementary Fig. 2a). For $m = n$, the wedges reduce to equilateral triangles whereas for $m = 2n$ they reduce

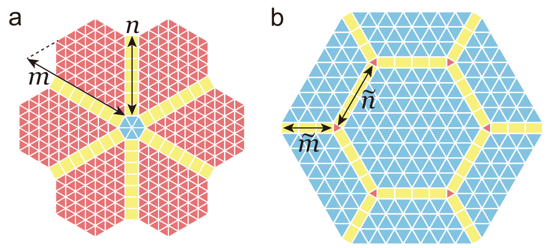

Supplementary Figure 2: Fundamental motifs of type IA and IB tilings (a and b, respectively), defining the two parameters of either tiling.

to $60° - 120°$ rhombi. These rhombi also represent the limiting case; tilings with m larger than $2n$, which are also possible, cannot be represented by fundamental motifs.

In Supplementary Fig. 2b we show the fundamental motif of type IB tiling with indicated $\widetilde{n}$ and $\widetilde{m}$. The central rosette of this motif is a hexagonal LT domain with $\widetilde{n}$ LT tiles along each edge, and each of the 6 trapezoid-shaped domains filling the gaps between the 6 radial spokes containing R tiles contains $\widetilde{m}$ rows of LT tiles.

Supplementary Note 2

Second-generation patterns

To complement the second-generation $k = 6$ type IA tiling in Fig. 1m of the main text, we display the second-generation $k = 9$ type IA, $k = 6$ type IB, and $k = 9$ type IB tilings in Supplementary Figs. 3, 4, and 5, respectively. In the $k = 6$ and $k = 9$ figures, the first-generation patterns magnified by factors $\beta_6 \approx 6.16228$ and $\beta_9 \approx 9.10977$, respectively, are plotted using dark blue lines.

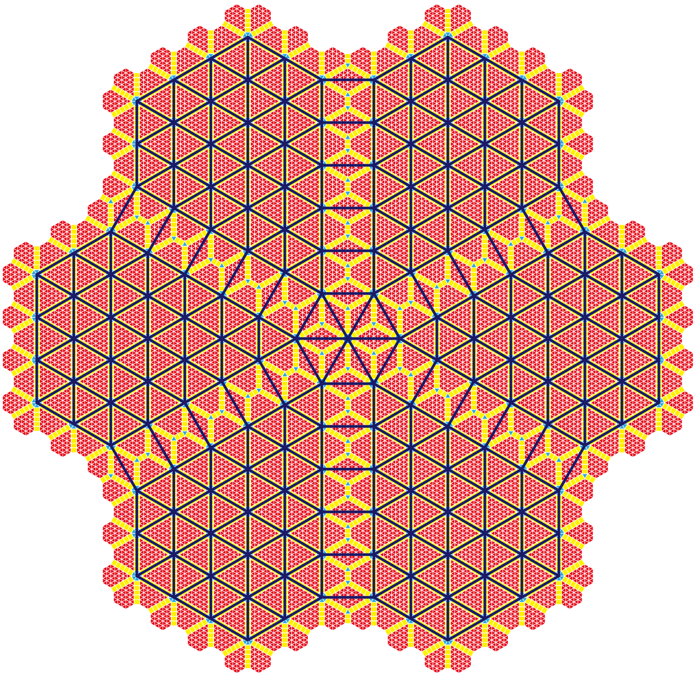

Supplementary Figure 3: Second-generation $k = 9$ type IA pattern; here the parameters of the subdivision rule from Fig. 1k in the main text are $n = 5$, $m = 7$. Overlaid is the first-generation pattern magnified by $\beta_9 \approx 9.10977$ (dark blue lines).

In Supplementary Fig. 3, we note the dominant triangular domains arranged in six diamond-shapes wedges stitched together along six radial seams. The patterns in Supplementary Figs. 4 and 5 can be viewed as a network consisting of three kinds of hexagonal domains containing LT tiles. The contour of the largest domains at the vertices of the first-generation pattern is a regular hexagon, whereas the contours of the other two domains seen in the center of the first-generation R and LT tile are not.

Apart from the symmetric flower-like patterns shown in Supplementary Figs. 3, 4, and 5 which illustrate the self-similar nature of our tilings, it is also instructive to examine their zoomed-in parts. Supplementary Figs. 6 and 7 present two such examples. The former figure contains the $k = 9$ type IA R tile after two subdivisions; the first-generation structure can still be easily recognized. This patch is dominated by (i) triangular domains consisting of the LT tiles. The other two types of domains—(ii) trapezoidal and (iii) diamond-shaped domains—constitute the roughly X-shaped cut across the rectangle, dotted by the single LT tiles plotted in blue.

In Supplementary Fig. 7, we zoom in on portions of the third-generation $k = 9$ type IB tiling. Here too there exist three types of LT-tile domains: (i) regular-hexagonal domains, (ii) domains with 3-fold symmetry and 3 short and 3 long sides, and (iii) domains with 2-fold symmetry and 2 short and 4 long side. The domain boundaries along the short and the long sides of the 2-fold and the 3-fold symmetric domains consist of 2 and 3 R tiles, respectively.

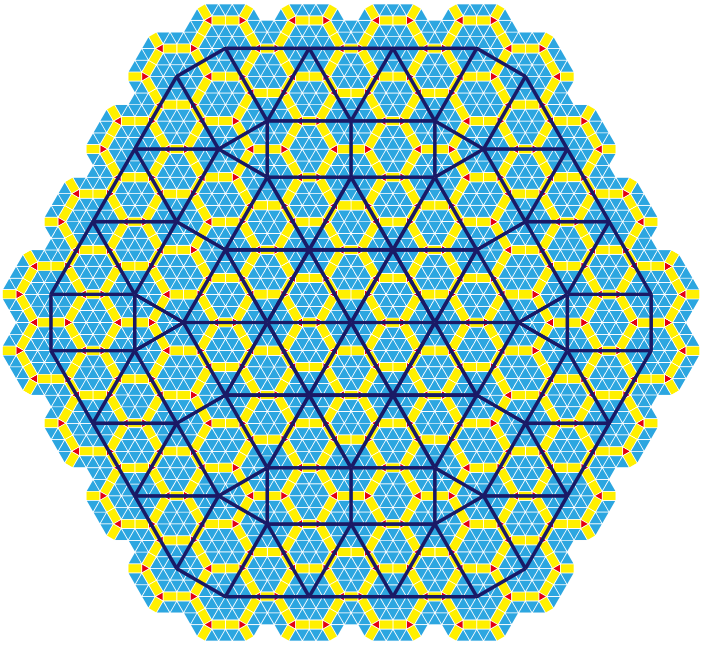

Supplementary Figure 4: Second-generation $k = 6$ type IB pattern; here the parameters of the subdivision rule from Fig. 1l in the main text are $\widetilde{n} = 2$, $\widetilde{m} = 1$. Overlaid is the first-generation pattern magnified by $\beta_6 \approx 6.16228$ (dark blue lines).

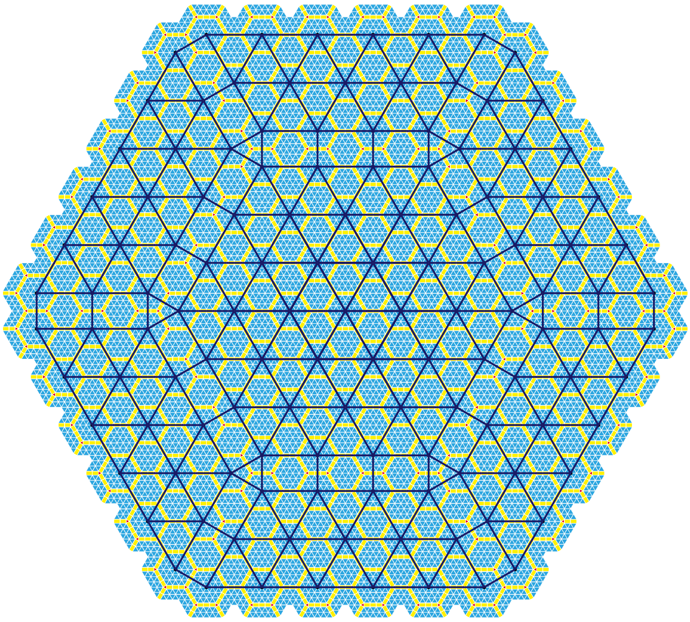

Supplementary Figure 5: Second-generation $k = 9$ type IB pattern; here the parameters of the subdivision rule from Fig. 1l in the main text are $\widetilde{n} = 3$, $\widetilde{m} = 2$. Overlaid is the first-generation pattern magnified by $\beta_9 \approx 9.10977$ (dark blue lines).

Yet their structure is somewhat more intricate and not as immediately appreciable than that of type IA tilings; it is perhaps best to consider it by examining the arrangement of the regular-hexagonal domains, which are generally separated from each other by the 2-fold and the 3-fold domains but also appear in pairs and in three-way triplets. Also instructive is viewing these patterns at an angle along one of the preferred directions and observing the zig-zagging of the domain boundaries along a given direction.

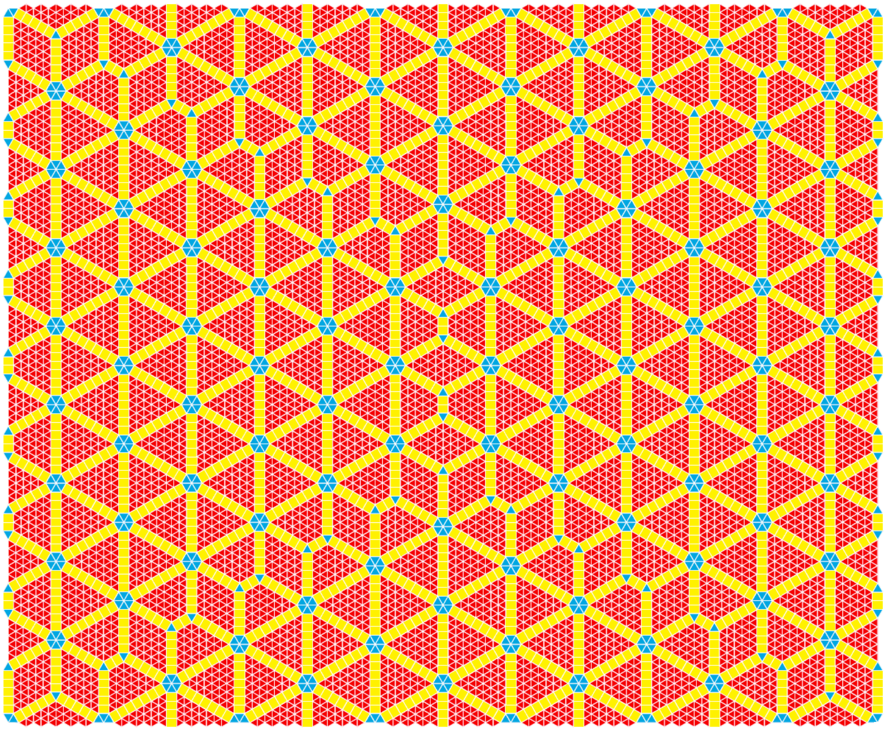

Supplementary Figure 6: Second-generation $k = 9$ R tile of type IA tiling.

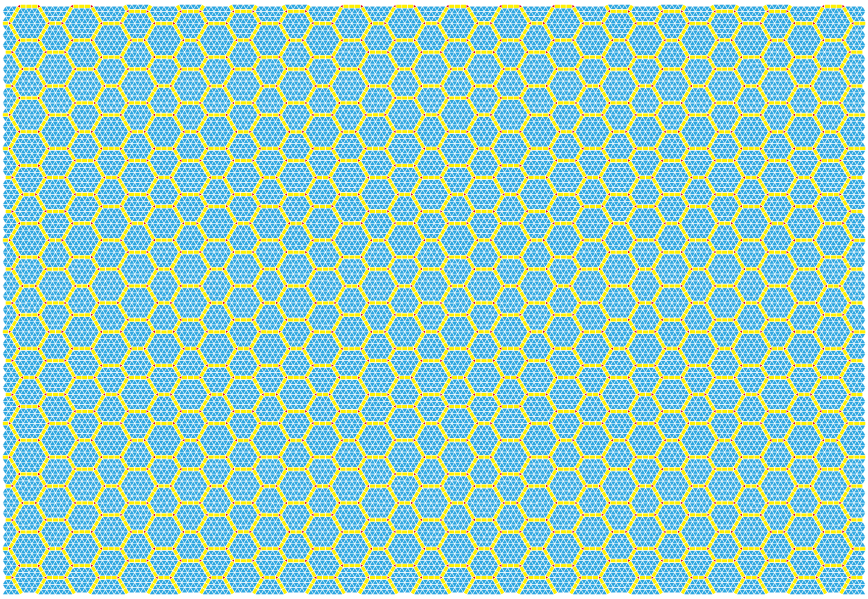

Supplementary Figure 7: Rectangular part of the $k = 9$ type IB tiling both showing an elaborate pattern consisting of three types of hexagonal domains.

Supplementary Note 3

Five $k = 6$ type I tilings

For $k = 6$, the identification of the different type I tilings described in the Methods section of the main text leads to the patterns shown in Supplementary Fig. 8. We first note that as shown in Supplementary Table I, the self-similar length ratios ϕ of type IC and ID tilings are smaller rather than larger than 1. This means that the physically longer length obeys the subdivision rule formally associated with the second row of the transformation matrix in Eq. (17) of the main text whereas the physically shorter length transforms according to the first row of the matrix. In Supplementary Fig. 8, we colored the ST and the LT tiles red and blue like in type IA, IB, and IE tilings where $\phi > 1$ for consistency. Secondly, when we construct the subdivision pattern for type IF tiling we recover the same pattern as in type ID tiling, which can be explained by the fact that type IF length ratio is reciprocal to that of type ID tiling. As a result, the type IF $\leftrightarrow$ type ID transformation involves a mere relabeling of the long and short edges without any physical difference.

The reader will notice that the inflation factors for type IA and IC tilings are also reciprocals so that a similar symmetry should also exist in these two patterns. Indeed, if one disregards the six rows of five ST tiles arranged around the very perimeter of type IA pattern, then type IA and IC fundamental motifs differ only in the arrangement of the very center and in the first annulus around it. In type IA, the center consists of six LT tiles and the first annulus contains six R tiles in the azimuthal orientation and six ST tiles, whereas in type IC the center is formed by six ST tiles and the first annulus consists of six R tiles in radial orientation separated by six LT tiles. This leads to two distinct sets of subdivision rules at the same length ratio. We thus conclude that there exist a total of 5 different $k = 6$ type I tilings.

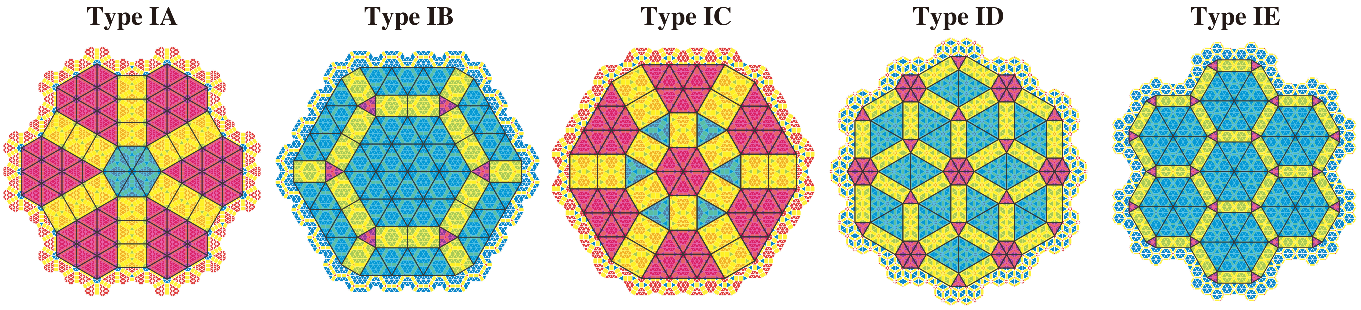

Supplementary Figure 8: Five second-generation $k = 6$ type I tilings. Type IA: $\alpha = 2, \beta = 3, \gamma = 1$, and $\delta = 4$, Type IB: $\alpha = 5, \beta = 1, \gamma = 2$, and $\delta = 1$, Type IC: $\alpha = 4, \beta = 1, \gamma = 3$, and $\delta = 2$, Type ID: $\alpha = 2, \beta = 1, \gamma = 3$, and $\delta = 4$, Type IE: $\alpha = 5, \beta = 2, \gamma = 1$, and $\delta = 1$.

Supplementary Table I: Six combinations of α, β, γ, and δ that all correspond to the $k = 6$ inflation factor. The table also includes the self-similar length ratio $\phi(\alpha, \beta, \gamma, \delta)$.

	α	β	γ	δ	$\phi(\alpha,\beta,\gamma,\delta)$
type ID	2	1	3	4	$\frac{-1+\sqrt{10}}{3\sqrt{3}} \approx 0.416$
type IA	2	3	1	4	$\frac{-1+\sqrt{10}}{\sqrt{3}} \approx 1.248$
type IC	4	1	3	2	$\frac{1+\sqrt{10}}{3\sqrt{3}} \approx 0.801$
type IF	4	3	1	2	$\frac{1+\sqrt{10}}{\sqrt{3}} \approx 2.403$
type IB	5	1	2	1	$\frac{2+\sqrt{10}}{2\sqrt{3}} \approx 1.490$
type IE	5	2	1	1	$\frac{2+\sqrt{10}}{3\sqrt{3}} \approx 2.980$

Supplementary Note 4

Higher-dimensional analysis

Lattice parameters

The values of the ratio of lattice constants, ℓ, the long-to-short length ratio ϕ, and the ratio of long and short edges ψ for type IA and type IB tilings are listed in Supplementary Tables II and III, respectively; for convenience, we list both the exact expressions and the approximate numerical values.

Supplementary Table II: Inflation factors, ratios of lattice constants a/c, ℓs, length ratios ϕ, and ratios of long and short edges ψ for type IA tilings with $k = 3, 6, 9, 12$, and ∞.

k	3	6	9	12	$\cdots$	∞
β_k	$\frac{3+\sqrt{13}}{2}$	$3+\sqrt{10}$	$\frac{9+\sqrt{85}}{2}$	$6+\sqrt{37}$	$\cdots$	∞
	3.303	6.162	9.110	12.083		
a/c	1	$\sqrt{3}$	$\sqrt{5}$	$\sqrt{7}$	$\cdots$	∞
	1	1.732	2.236	2.646	$\cdots$	∞
ℓ	$\frac{\sqrt{39}+\sqrt{3}}{6}$	$\frac{\sqrt{10}-1}{3}$	$\frac{\sqrt{51}-\sqrt{15}}{6}$	$\frac{\sqrt{777}-4\sqrt{21}}{21}$	$\cdots$	0
	1.330	0.721	0.545	0.454		
ϕ	$\frac{\sqrt{39}+\sqrt{3}}{6}$	$\frac{\sqrt{30}-\sqrt{3}}{3}$	$\frac{\sqrt{255}-5\sqrt{3}}{6}$	$\frac{\sqrt{111}-4\sqrt{3}}{3}$	$\cdots$	$\frac{2\sqrt{3}}{3}$
	1.330	1.248	1.218	1.202	$\cdots$	1.155
ψ	$\frac{\sqrt{39}+\sqrt{3}}{6}$	$\frac{\sqrt{30}-\sqrt{3}}{9}$	$\frac{\sqrt{255}-5\sqrt{3}}{30}$	$\frac{\sqrt{111}-4\sqrt{3}}{21}$	$\cdots$	0
	1.330	0.416	0.244	0.172		

Supplementary Table III: Inflation factors, ratios of lattice constants c/a, ℓs, length ratios ϕ, and ratios of long and short edges ψ for type IB tilings with $k = 3, 6, 9, 12$, and ∞.

k	3	6	9	12	$\cdots$	∞
β_k	$\frac{3+\sqrt{13}}{2}$	$3+\sqrt{10}$	$\frac{9+\sqrt{85}}{2}$	$6+\sqrt{37}$	$\cdots$	∞
	3.303	6.162	9.110	12.083		
c/a	1	$\sqrt{2}$	$\sqrt{3}$	2	$\cdots$	∞
	1	1.414	1.732	2	$\cdots$	∞
ℓ	$\frac{\sqrt{39}+\sqrt{3}}{6}$	$\frac{\sqrt{15}+\sqrt{6}}{3}$	$\frac{\sqrt{85}+7}{6}$	$\frac{\sqrt{111}+5\sqrt{3}}{6}$	$\cdots$	∞
	1.330	2.107	2.703	3.199		
ϕ	$\frac{\sqrt{39}+\sqrt{3}}{6}$	$\frac{\sqrt{30}+2\sqrt{3}}{6}$	$\frac{\sqrt{255}+7\sqrt{3}}{18}$	$\frac{\sqrt{111}+5\sqrt{3}}{12}$	$\cdots$	$\sqrt{3}$
	1.330	1.490	1.561	1.600	$\cdots$	1.732
ψ	$\frac{\sqrt{39}+\sqrt{3}}{6}$	$\frac{\sqrt{30}+2\sqrt{3}}{3}$	$\frac{\sqrt{255}+7\sqrt{3}}{6}$	$\frac{\sqrt{111}+5\sqrt{3}}{3}$	$\cdots$	∞
	1.330	2.980	4.682	6.399		

Physical-space and reciprocal-space basis vectors

The physical-space basis vectors for type IA and type IB tilings with $k = 3, 6, 9, 12$, and ∞ are shown in Supplementary Fig. 9. At $k = 3$ corresponding to the bronze-mean tiling, the four basis vectors are evidently all independent which is witnessed by the non-integer ratio of their lengths given by Eq. (12) of the main text; at $k = 3$,

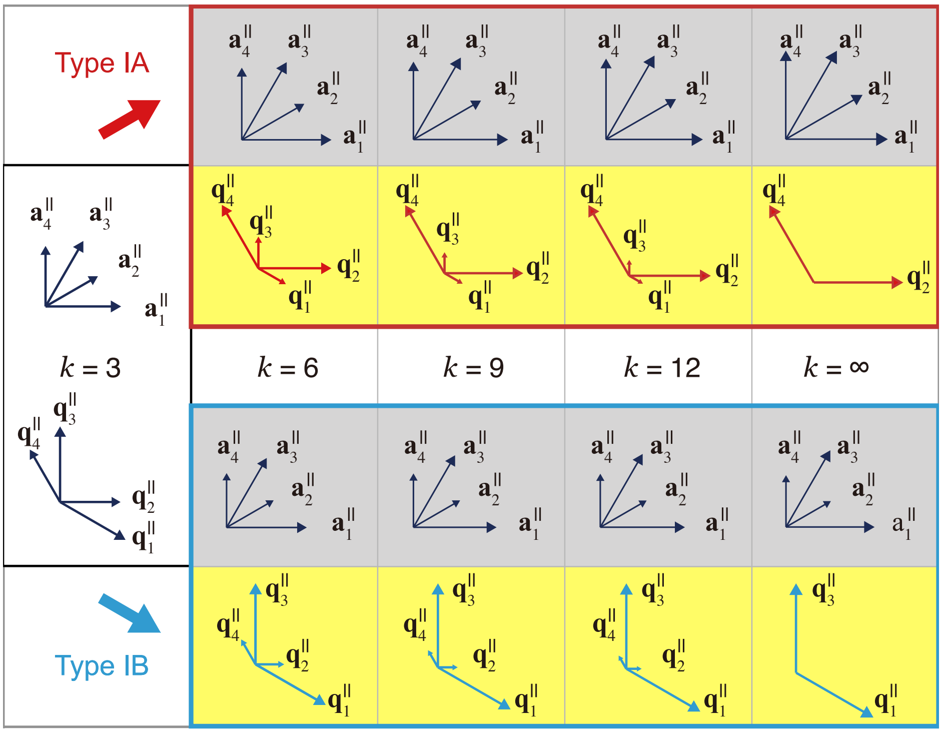

Supplementary Figure 9: Physical-space basis vectors $\mathbf{a}_j^{||}$ $(j = 1, 2, 3, 4)$ and reciprocal-space basis vectors $\mathbf{q}_j^{||}$ $(j = 1, 2, 3, 4)$ for $k = 3, 6, 9, 12$ and ∞ type IA and IB tilings. As k is increased, the four physical-space basis vectors become increasingly less linearly independent and for $k \to \infty$, only two of them are. Concomitantly, the magnitudes of vectors $\mathbf{q}_1^{||}$ and $\mathbf{q}_3^{||}$ in type IA tilings and the magnitudes of vectors $\mathbf{q}_2^{||}$ and $\mathbf{q}_4^{||}$ in type IB tilings decrease; in the limit $k \to \infty$, these four vectors vanish.

$$\left|\frac{\mathbf{a}_{\text{odd}}^{||}}{\mathbf{a}_{\text{even}}^{||}}\right| = \frac{\sqrt{39}+\sqrt{3}}{6} \approx 1.330. \tag{1}$$

As k is increased, this ratio in the type IA tilings approaches

$$\lim_{k\to\infty} \frac{a\ell}{c} = \lim_{k\to\infty} \frac{-k+4+\sqrt{k^2+4}}{2\sqrt{3}} = \frac{2}{\sqrt{3}}, \tag{2}$$

where we used Eqs. (13) and (14) of the main text. As the basis vectors are 30° apart, when $k \to \infty$ then $\mathbf{a}_1^{||}+\mathbf{a}_3^{||} = 2\mathbf{a}_2^{||}$ and $\mathbf{a}_1^{||} + 2\mathbf{a}_4^{||} = 2\mathbf{a}_3^{||}$ etc. so that the four basis vectors $\mathbf{a}_1, \mathbf{a}_2, \mathbf{a}_3$, and $\mathbf{a}_4$ are no longer independent from each other. As a result, only two basis vectors are needed to describe the tiling, which indicates translational periodicity; the rotational symmetry is evidently six-fold. In type IB tilings at $k \to \infty$, $\mathbf{a}_2^{||} + \mathbf{a}_4^{||} = \mathbf{a}_3^{||}$ etc., which leads to the same conclusion.

Supplementary Fig. 9 also shows the reciprocal-space basis vectors, which too reflect the convergence towards the hexagonal lattice as k is increased. This is witnessed by the increasingly smaller magnitude of $\mathbf{q}_1^{||}$ and $\mathbf{q}_3^{||}$ in type IA tiling and $\mathbf{q}_2^{||}$ and $\mathbf{q}_4^{||}$ in type IB tiling.

Supplementary Note 5

Self-similarity in diffraction patterns

To find the metallic means in diffraction images in experiments, it is helpful to use the following identities pertaining to the ratio of long and short edges: $\sqrt{3}/\psi+2 = \beta_k$ (type IA) and $\sqrt{3}\psi+1 = \beta_k$ (type IB). These identities imply that for example in the $k = 6$ type IB pattern in Fig. 3b in the main text or in the right panel of Supplementary Fig. 10,

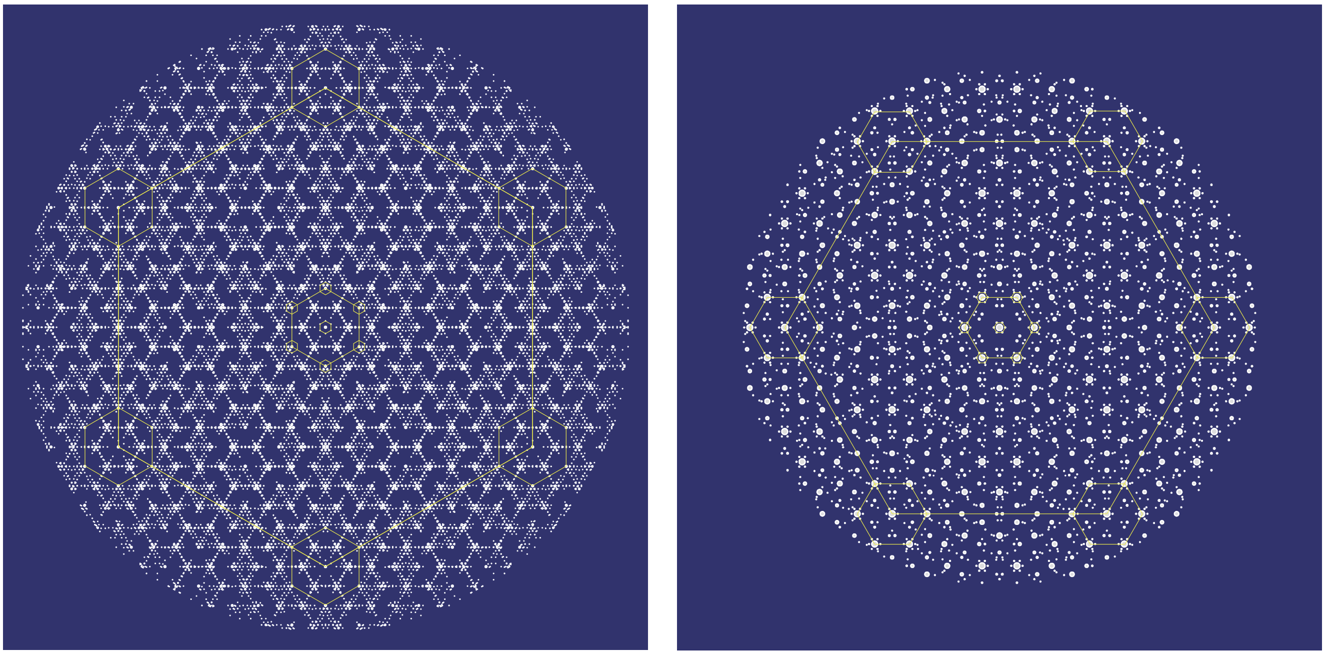

Supplementary Figure 10: Extended areas of the Fourier transforms of $k = 6$ type IA and IB tilings (left and right panel, respectively), with the yellow hexagons of three different sizes emphasizing the presence of the sixth metallic mean.

the intensities of the small six satellite peaks forming a hexagon around the pronounced peak with indices $(2, 1, 1, 0)$ are scaled by a factor of $1/\beta_6$.

Extended areas of $k = 6$ Fourier transforms of type IA and IB tilings are shown in Supplementary Fig. 10. To demonstrate the presence of the sixth metallic mean, we drew a few hexagons characterizing the pattern of three different sizes (yellow lines); on going form the smallest to the middle and from the middle to the largest hexagon, the linear dimension is increased by a factor of the sixth metallic mean $3 + \sqrt{10} \approx 6.162$.

Supplementary Note 6

Inflation factors

In Supplementary Tables IV and V, we list the inflation factors of type IB and type IIA tilings

$$\lambda_+^{\mathrm{IB}} = \frac{\widetilde{m} + 1 + 2\widetilde{n} + \sqrt{(\widetilde{m} - 1)^2 + 4\widetilde{n}(2 + \widetilde{m} + \widetilde{n})}}{2}, \tag{3}$$

and

$$\lambda_+^{\mathrm{IIA}} = \frac{\sqrt{3}(n + 1) + \sqrt{3(n - 1)^2 + 8m}}{2}, \tag{4}$$

respectively, for the physically meaningful combinations of n and m or $\widetilde{n}$ and $\widetilde{m}$ where n and $\widetilde{n} \leq 5$. These tables complement Table I of the main text.

Interestingly, if $\widetilde{n} = \widetilde{m}$ then the inflation factor of the type IB tiling is an integer. These special cases correspond to periodic-crystalline rather than quasicrystalline tilings as illustrated by the $\widetilde{n} = \widetilde{m} = 2$ type IB tiling encoded by the subdivision patterns in Supplementary Fig. 11. Supplementary Fig. 12 shows the second-generation $\widetilde{n} = \widetilde{m} = 2$ type IB tiling both without and with the outline of the first-generation tiling; the former more evidently shows the periodic-crystalline nature of the pattern. In this type IB tiling, the ratio of long and short lengths ϕ is $\sqrt{3}$ and the inflation factor is 7.

The periodic-crystalline nature of the $\widetilde{n} = \widetilde{m}$ type IB tiling is exact as the tiling consists of a single hexagonal domain evident in the left panel of Supplementary Fig. 12. Yet the existence of the periodic-crystalline $\widetilde{n} = \widetilde{m}$ type IB

Supplementary Table IV: Inflation factors of type IB tilings; the multiple-of-3 metallic-mean inflation factors are typeset in boldface.

	$\widetilde{n}$				
$\widetilde{m}$	1	2	3	4	5
0	$\boldsymbol{\frac{3+\sqrt{13}}{2}}$	$\frac{5+\sqrt{33}}{2}$	$\frac{7+\sqrt{61}}{2}$	$\frac{9+\sqrt{97}}{2}$	$\frac{11+\sqrt{141}}{2}$
1	4	$\boldsymbol{3+\sqrt{10}}$	$4+3\sqrt{2}$	$5+2\sqrt{7}$	$2\left(3+\sqrt{10}\right)$
2	$\frac{5+\sqrt{21}}{2}$	7	$\boldsymbol{\frac{9+\sqrt{85}}{2}}$	$\frac{11+\sqrt{129}}{2}$	$\frac{13+\sqrt{181}}{2}$
3	$3+\sqrt{7}$	$4+\sqrt{15}$	10	$\boldsymbol{6+\sqrt{37}}$	$7+\sqrt{51}$
4	$\frac{7+\sqrt{37}}{2}$	$\frac{9+\sqrt{73}}{2}$	$\frac{11+3\sqrt{13}}{2}$	13	$\boldsymbol{\frac{15+\sqrt{229}}{2}}$
5	$2\left(2+\sqrt{3}\right)$	$5+\sqrt{22}$	$6+\sqrt{34}$	$7+4\sqrt{3}$	16

Supplementary Table V: Inflation factors of type IIA tiling represented by the subdivision patterns in Fig. 5c of the main text.

	n				
m	1	2	3	4	5
n	$\sqrt{3}+\sqrt{2}$	$\frac{3\sqrt{3}+\sqrt{19}}{2}$	$2\sqrt{3}+3$	$\frac{5\sqrt{3}+\sqrt{59}}{2}$	$3\sqrt{3}+\sqrt{22}$
$n+1$	$\sqrt{3}+2$	$3\sqrt{3}$	$2\sqrt{3}+\sqrt{11}$	$\frac{5\sqrt{3}+\sqrt{67}}{2}$	$3\sqrt{3}+2\sqrt{6}$
$n+2$	$\sqrt{3}+\sqrt{6}$	$\frac{3\sqrt{3}+\sqrt{35}}{2}$	$2\sqrt{3}+\sqrt{13}$	$5\sqrt{3}$	$3\sqrt{3}+\sqrt{26}$
$n+3$	$\sqrt{3}+2\sqrt{2}$	$\frac{3\sqrt{3}+\sqrt{43}}{2}$	$2\sqrt{3}+\sqrt{15}$	$\frac{5\sqrt{3}+\sqrt{83}}{2}$	$3\sqrt{3}+2\sqrt{7}$
$n+4$	$\sqrt{3}+\sqrt{10}$	$\frac{3\sqrt{3}+\sqrt{51}}{2}$	$2\sqrt{3}+\sqrt{17}$	$\frac{5\sqrt{3}+\sqrt{91}}{2}$	$3\sqrt{3}+\sqrt{30}$
$n+5$	$3\sqrt{3}$	$\frac{3\sqrt{3}+\sqrt{59}}{2}$	$2\sqrt{3}+\sqrt{19}$	$\frac{5\sqrt{3}+3\sqrt{11}}{2}$	$3\sqrt{3}+4\sqrt{2}$
$n+6$	$\sqrt{3}+\sqrt{14}$	$\frac{3\sqrt{3}+\sqrt{67}}{2}$	$2\sqrt{3}+\sqrt{21}$	$\frac{5\sqrt{3}+\sqrt{107}}{2}$	$3\sqrt{3}+\sqrt{34}$

tiling appears to be accidental as the inflation factor is a single integer; usual periodic crystals are characterized by arbitrary integer inflation factors (except, of course, 1). This conjecture is supported by the $n=2, m=3$ and $n=4, m=6$ type IA tilings which too have integer inflation factors (5 and 8, respectively, as seen in Table I of the main text) but are not periodic-crystalline.

These arguments suggest the $\widetilde{n}=\widetilde{m}$ type IB tilings may be referred to as accidental periodic crystals. We anticipate that type IIB tilings with $\widetilde{n}=\widetilde{m}$ (concisely represented by the subdivision pattern of the R tile in Fig. 5c of the main text) may too be accidental periodic crystals in the same sense as $\widetilde{n}=\widetilde{m}$ type IB tilings, consisting of regular hexagonal domains of ST tiles separated by domain walls of R tiles in the radial orientation relative to the domains and LT tiles.

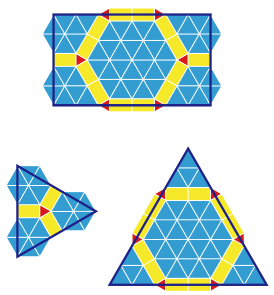

Supplementary Figure 11: Subdivision patterns for the R, ST, and LT type IB tiles for $\widetilde{n}=\widetilde{m}=2$, which corresponds to a periodic-crystalline tiling.

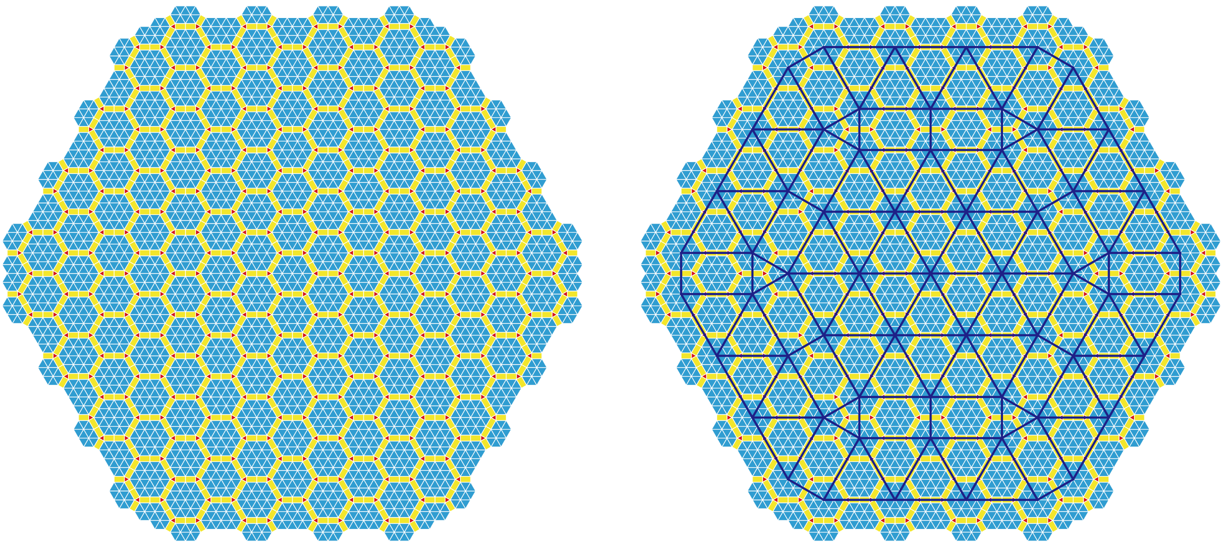

Supplementary Figure 12: Second-generation $\widetilde{n} = \widetilde{m} = 2$ type IB pattern (left; the right panel is the same pattern with the first-generation tiling outlined in dark blue).
